# Applications of synergetics in psychology: interpersonal synchrony in social systems

**DOI:** 10.3389/fnetp.2025.1739213

**Published:** 2026-01-08

**Authors:** Wolfgang Tschacher

**Affiliations:** University Hospital of Psychiatry and Psychotherapy, University of Bern, Bern, Switzerland

**Keywords:** coordination dynamics, gestalt psychology, network physiology, pattern formation, pro-sociality, psychotherapy, self-organization, surrogate synchrony (SUSY)

## Abstract

The Haken-Kelso-Bunz paradigm of motor coordination has instigated experimental research on pattern formation with a focus on body movement in intra- as well as interpersonal contexts. The current research on interpersonal synchrony in psychology can be seen to generalize on this initial synergetic approach. A large body of evidence has been aggregated to date showing that synchrony is a common signature of social systems as studied in psychotherapy research, in social psychology and in the dynamics of large groups. Interestingly, such synchronization processes occur spontaneously, generally outside the awareness of the individuals involved in them. Novel qualities arise due to interpersonal synchrony, which is reminiscent of self-organization as conceived by Haken’s Synergetics. The degree of synchrony of physiological and behavioral processes was often found associated with cognitive and emotional variables and is thus considered an important aspect of ‘embodied cognition’. Therefore, synchrony additionally points to circular causality in mind-body relations and throws a light on the synergetic slaving principle in psychology.

## 1 Introduction

**FIGURE 1:**
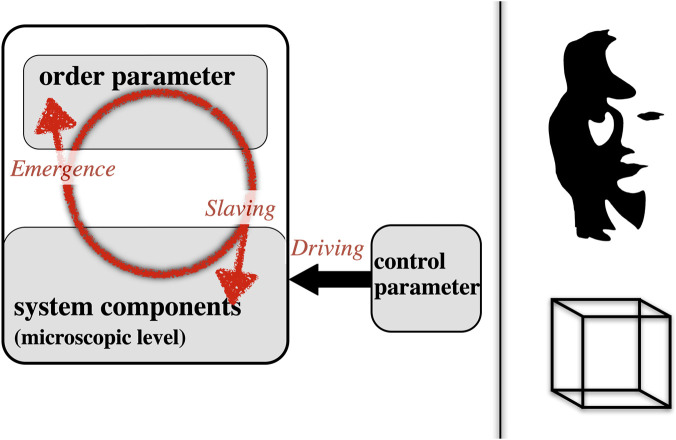
Left panel: Schema of basic synergetic concepts (see text). Right panel: two examples of Gestalt displays (top: Wikimedia commons, Mooney, 1957; bottom: Necker cube).

The core phenomenon of Synergetics is the self-organization of complex open systems, where systems may consist of numerous components (the microscopic level of system complexity), from whom an emergent pattern (the order parameter, the macroscopic level of the system) arises. The system components may initially show disorganized and uncorrelated behavior (thus, have many degrees of freedom) that due to the influence of the emerging order parameter becomes increasingly organized and synchronized. This process of increased organization of system components was termed the slaving principle (Haken, 1977). Importantly, such organization processes were considered *self*-organized since no external agent was responsible for governing the pattern formation. In addition, the pattern described by the order parameter has the property of asymptotic stability, hence it is an attractor that upholds the pattern in the face of perturbations. The external impact necessary for having the order parameter arise was unspecific, and is in general (in physical systems) provided by an external source of energy called the control parameter. These fundamental concepts of Synergetics are depicted in schematic form in Figure 1 on the left-hand side.

Hermann Haken, the founder of Synergetics, emphasized the decidedly interdisciplinary nature of his theory: The synergetic conceptualization rests on mathematics and is thus open for applications in any field of science. Hence, Synergetics is a structural theory with a focus on pattern formation processes in whatever systems. Whereas initially the study objects of Synergetics were complex systems in physics such as laser light or fluid dynamics, the interest soon widened to encompass pattern formation also in physiology, sociology, and psychology. The former extension of Synergetics (Haken and Koepchen, 1991) motivated studies in the field of network physiology (Ivanov, 2021) and applications in medicine and oncology (Sawicki et al., 2022; Costa et al., 2025). The application of synergetic principles to cognition (Haken and Stadler, 1990) was supported by the Gestalt tradition of psychology that had been a leading theory in academic psychology of the early 20th century and was then again invigorated by Synergetics (Tschacher, 1997).

In terms of Figure 1, what are the analog psychological concepts of Gestalt psychology for ‘order parameter’, ‘system components’ and ‘control parameter’? On the right side of Figure 1, two well-known Gestalt displays are shown, the bistable Necker cube (bottom) and a bistable Mooney face that can also be viewed as a man playing the saxophone. The order parameter is formed as soon as one of the Gestalt perceptions is realized (either the face or the sax player; and one of the two 3D percepts of the Necker cube). The system components are the elements of the cube, i.e., lines and angles, and respectively the black areas of the Mooney figure. The control parameter in psychological terms is motivational, namely, the law of closure (*Prägnanzgesetz*) of Gestalt theory, which is a motivational tendency to detect a meaningful whole rather than just a set of elements (Koffka, 1935). In a general application of Synergetics to psychology (Tschacher and Haken, 2007), we found the motivation of avoiding ‘cognitive dissonance’ (Festinger, 1964) and the ecological-psychology concept of ‘affordance’ (Gibson, 1979; Bruineberg and Rietveld, 2014) may have the function of control parameters in psychological systems. Both dissonance and affordance were originally developed by Gestalt psychology (Lewin, 1936). As Friston, 2017 noted, there is also a close connection between his neuroscientific free-energy principle and the circular causality of Synergetics.

Further examples of self-organized pattern formation were described for overt behavior. Haken’s cooperation with the neuroscientist Scott Kelso concerned the rhythmic coordination of body limbs such as the index fingers of both hands of an individual instructed to move both fingers at the speed of the experimenter’s metronome. One result was that finger movements spontaneously synchronize, and that two patterns of movement coordination were observed, either parallel or anti-parallel movement (the Haken-Kelso-Bunz model: Haken et al., 1985; Mechsner and Prinz, 2003). It was found to be almost impossible for participants to move the fingers at a certain frequency but ‘out of sync’, hence resisting synchronization (Uccelli et al., 2025); this indicates the presence of a synchrony attractor. The patterns, i.e., the order parameters, depended on the control parameter, here the frequency prescribed by the metronome; the initial parallel movement necessarily switched to the anti-parallel pattern at a certain critical frequency. Interestingly, such coordination dynamics was also found in interpersonal contexts (Schmidt and O'Brien, 1997; Knoblich et al., 2011) where participants synchronized their movements spontaneously even in the absence of the intention or instruction to coordinate, pointing to emergent coordination in joint action. The phase transitions predicted by synergetic theory, thus the transitions between the parallel pattern (phase angles approximately zero degrees) and anti-parallel pattern (phase angles around 180°), were observed not only in movement coordination within an individual but also in interpersonal experiments (Kelso, 1995).

The goal of the present perspective article is to review the findings of the past two decades that studied the phenomenon of interpersonal synchrony in naturalistic contexts and with an emphasis on ecological validity. Intra- and interpersonal coordination dynamics was initially investigated using experiments in the laboratory, where participants were instructed to move fingers, arms, or legs according to experimenters’ instructions, directed by metronomes or other timing devices. In other words, such experimental setups were rather artificial (“please drum together as you think best”) and obviously afforded the hypothesized synchronized behavior in the first place. The next research questions of Synergetics-informed psychology became thus: Does the Haken-Kelso-Bunz model extend into daily life, into social interaction? If yes, are there synchronized patterns other than movement synchrony, such as synchrony of physiological measures? Are mental and emotional consequences linked with being in- or out-of-sync?

## 2 Interpersonal synchrony

### 2.1 Methods for the capture of movement and physiology

Studying synchrony ‘in the wild’ called for a number of methodological adaptations. First, how may we detect the amount of body movement under naturalistic conditions in an unobtrusive manner? There was a demand to study movement synchrony between therapists and clients with the goal of contributing to psychotherapy process research. In many institutions and therapy ambulatories, sessions are routinely recorded on video, for later use in supervision. Grammer et al. (1999) described a method by which body movement can be extracted from video by way of counting the pixel changes (the “motion energy”) in different regions of interest in the video. Thus, the motion energies of therapist and client, both depicted on routine video recordings, constitute a shortcut to time series of both persons’ body movement. We developed an application for motion energy analysis, the MEA app (Ramseyer and Tschacher, 2011; Ramseyer, 2020). New technology also simplified the study of physiological signals, such as heart rate and other cardiac measures, skin conductance responses, and respiratory measures. Physiological time series are derived from wearable devices with integrated non-invasive sensors (e.g., Sun et al., 2023).

### 2.2 Methods for the estimation of synchrony

Motion capture apps and wearable devices provide the raw data for the computation of synchrony based on (commonly dyadic) time series measured simultaneously. These time series may cover various time scales, ranging from close to 1 hour (the duration of a psychotherapy session) to several minutes (the duration of brief conversations) and even shorter momentary interactions. The algorithms used for synchrony computation have different mathematical and statistical foundations and are sometimes inconsistent as to the resulting synchronies they suggest (Altmann et al., 2022). Most research to date has applied time-based algorithms that implement the cross-correlation function of two time series to estimate their synchrony (e.g., surrogate synchrony, SUSY: Tschacher, 2023). A similar ‘concordance’ approach uses the correlation between local slopes of the two time series as the definition of synchrony (e.g., surrogate concordance, SUCO: Tschacher and Meier, 2020). Frequency-based algorithms assess synchrony by cross-wavelet coherence, a method related to the Fourier decomposition (e.g., Fujiwara and Daibo, 2018). Finally, there are approaches based on information theory such as mutual information and the quantification of cross-recurrence (Coco and Dale, 2014). The following overview, however, will predominantly concern time-based cross-correlation approaches of synchrony, which by integration of surrogate analysis can generate an effect size for the degree of synchronization of each dyadic time series, thus each system (Ramseyer and Tschacher, 2010). Analysis of the lag structure of the cross-correlation function also allows to detect the leader or follower in the respective process, i.e., which of the two time series (e.g., the therapist’s or the client’s) is active prior to the other, hence showing signatures of Granger causality.

### 2.3 Synchrony in psychotherapy sessions

Early psychotherapy research was focused on the reduction of degrees of freedom as the core phenomenon of Synergetics, thus assessing the pattern formation that was assumed to occur in courses of psychotherapy from session to session. Tschacher and Grawe (1996) analyzed 22 psychotherapy courses whose sessions were fully rated using a set of rating scales at the termination of each session. Using principal component analyses (Cattell’s O technique), we found that the degrees of freedom, operationalized by the number of components with eigenvalues exceeding 1, became clearly reduced in the course of sessions, pointing to the hypothesized self-organization process in therapy systems. This study was later replicated in a larger dataset and by implementation of a further measure of order, Banerjee’s ‘order omega’ (Banerjee et al., 1990; Tschacher et al., 1998).

A large study (Ramseyer and Tschacher, 2011) was based on 15-min excerpts of 104 psychotherapy sessions conducted in a university outpatient clinic. Thus, the focus changed from synchrony between multiple sessions to the synchrony within a session. MEA of video recordings generated time series with the sampling rate of the underlying videos (here, 10 Hz), which were then analyzed by surrogate synchrony using absolute cross-correlations. Synchrony significantly exceeded the control condition of surrogate synchronies, and significantly predicted patient’s assessments of therapeutic alliance after the respective sessions. Synchrony was also found predictive of overall therapy outcome at the end of all sessions, and was negatively associated with patients’ symptom load. This study was influential in that it instigated synchrony research by numerous other researchers (e.g., Seikkula et al., 2015; Paulick et al., 2018) and subsequent meta-analyses (Wiltshire et al., 2020; Atzil-Slonim et al., 2023; Gregorini et al., 2025). Much of this research assessed synchrony based on interlocutors’ body movement derived from video, yet increasingly this ‘MEA’ approach was complemented by physiological measurements.

An isolated early study of “interpersonal physiology” used synchrony defined as the correlation of therapist’s and patient’s heart rates (HR), finding links with tension and antagonism in a case study (Dimascio et al., 1957). Our group initiated a study on physiological synchrony in 55 psychotherapy sessions with a monitoring of HR, heart rate variability (HRV), and respiration behavior (RESP) in the 1990s (Tschacher, 1997; Section 8.6). Complete results of this trial were only published much later, with the availability of more sophisticated algorithms SUSY and SUCO (Tschacher and Meier, 2020). Both algorithms allowed a distinction between in-phase and anti-phase synchrony based on cross-correlations not set to absolute values. Significant synchrony was found for HR (anti-phase), HRV (significant only for absolute cross-correlations) and RESP (in-phase). The electrocardiograms, thus the exact timing of heart beats, were not synchronized. Several associations were found between synchronies and alliance ratings and client’s wellbeing.

Electrodermal synchrony was the focus of a study of 21 courses of cognitive-behavioral psychotherapy with altogether 299 monitored sessions (Tschacher et al., 2025). Therapist-patient synchronies of skin-conductance response, hence sympathetic activation, was significantly given in SUSY, and was in-phasic. The extent of synchrony was associated with patients’ emotional positivity and attenuated emotional arousal. Concerning the auto-correlation function underlying SUSY, the function was asymmetric, as patients’ leading roles were more pronounced than therapists’. Patients’ leading was associated with better therapeutic alliance/bond. Recent reviews of the field of physiological synchrony, however, point to findings that vary considerably, ranging from significant to no associations between synchrony and therapy outcome and alliance (Jennissen et al., 2024; Kleinbub, 2017).

### 2.4 Synchrony in multi-person systems

Synchrony is not restricted to dyadic social systems. There are many examples for pattern formation in groups, crowds (Strogatz et al., 2005; Ardizzi et al., 2020), and masses (Freud, 1921). Early on, Synergetics had already considered societal levels of self-organization, focusing on public opinion as a kind of pattern formation (Haken, 1984). To extend the SUSY method to larger social systems, our group developed and tested a multivariate algorithm based on correlation matrices with surrogate tests (mvSUSY, Meier and Tschacher, 2021). An alternative method is to compute all dyadic permutations of the components/persons of the multi-person system and compute an aggregate synchrony on the system level. This method was chosen in a number of studies to assess the overall synchrony of multi-person audiences in concerts; results pointed to significant synchrony of movement and various physiological signals (HR, HRV, skin-conductance response, respiration rate) among the members of audiences listening to music presentations; the individual contributions to audience synchrony were associated with aesthetic experiences (Tschacher et al., 2024). Multi-person systems are likewise relevant in group psychotherapy and couple therapy. Couple therapy sessions with two therapists and two spouses were analyzed in the context of the ‘Relational Mind’ project (Karvonen et al., 2016). It is too early for generalized findings since considerable differences were found, e.g., between movement synchrony and physiological synchrony of the same sessions (Kykyri et al., 2025) and between the synchronies of subsystems (client-client, client-therapist, therapist-therapist: Coutinho et al., 2025).

### 2.5 Synchrony in conversations

Research on synchrony also concerned natural discussions and conversations. A study with 84 unacquainted dyads (Tschacher et al., 2014), who discussed topics drawn from an urn, implemented three conditions: a cooperative instruction, a competitive instruction, and a humorous task (“compose a menu of five courses, each of which you definitely find disgusting”). Participants were generally informed that the study goal was to observe interaction processes in general, “synchronization” or “nonverbal” was not mentioned. Synchrony was based on both participants’ whole-body movement assessed using MEA and was found significantly present, with the fun task creating clearly elevated levels and the cooperation task unexpectedly the lowest level. Synchrony was linked with subsequent positive affect.

Studies have also been conducted with the goal of characterizing mental disorders. In a trial with 27 remitted schizophrenia patients, video data of role-play episodes of a therapist and the patients were reanalyzed to assess movement synchrony and the respective leading or following roles (Kupper et al., 2015). As a result, synchrony was linked with the symptomatology of patients; overall, more severe symptoms and low social functioning entailed lowered nonverbal synchrony. The symptom domains were specifically linked with patients’ following role (less following when patients’ negative symptoms and depression were high). Therapists’ following was attenuated with higher florid symptoms and cognitive disorganization of patients. Georgescu et al. (2020) studied dyadic interactions to assess the differences between dyadic movement synchrony in three dyad types: Both participants diagnosed with (high-functioning) autism spectrum disorder (ASD), both participants non-ASD, and a (MIXED) group. Using an approach similar to Tschacher et al.'s (2014), it was found that the non-ASD dyads showed significantly higher synchrony than both the MIXED and ASD dyads, which did not differ from each other. The effect was not mediated by different amounts of nonverbal movement, thus not merely a result of lowered expressions of ‘body language’ in autism.

## 3 Discussion

This article offers a perspective on the impact that Haken’s Synergetics has on psychological research, fostering an understanding specifically of pattern formation processes within clinical and social psychology, termed ‘synchrony’ by a majority of researchers. Terminology has however not fully stabilized in this fast-growing field of psychology, thus what I call synchrony here may also come under the flag of ‘mimicry’, ‘entrainment’, ‘coordination’, ‘attunement’, ‘resonance’, ‘contagion’ etc. Some of these terms have an intentional flavor of one person imitating the other, whereas it is important to clarify that such social pattern formation is a spontaneous process, thus is self-organized and non-intentional. In naturalistic contexts outside the lab, people get synchronized without wishing to synchronize, often without any awareness of their being and becoming ‘in-sync'.

Almost all the studies that have used serious methods and algorithms for assessing synchrony have found reliable evidence of the phenomenon *per se*. Findings pointing to the existence of synchrony above random coincidence have been based on a large range of processes: various peripheral physiological and central-nervous measures, body movement, facial expression, prosodic variables, lexicon, body posture, and more. Such measures are typically grounded in bodily, objective data. The ensuing question is, of course, what is the function of such synchrony? Which mental, subjective, and experiential variables are associated with synchrony?

The frequent answer, yet the not-so-frequent finding, considers synchrony as the prosocial glue in social interactions, linked with empathy, therapeutic alliance and bond in psychotherapy. In this view, synchrony constitutes an implicit signature of interpersonal understanding and a common factor of therapeutic effectiveness. Yet, the research to date did not always support the hypothesized pro-sociality of synchrony. There are several possible reasons for this preliminary unstable result.

First, the field has not reached a standardization of the algorithms to be used. Different algorithms generate different results, as is well known (Altmann et al., 2022). Even if the same methodology is implemented, parameter settings are crucial. This is evident for one of the best-known approaches, windowed cross-correlation, where *not* using absolute correlation values as the standard can be a game-changer. In the case of the study of Tschacher et al. (2014), for instance, re-analysis without absolutes showed that the interactions in the competition condition was highly synchronized, but in anti-phase.

Second, synchrony depends on the context. As seen in this re-analysis, competition and conflict is often associated with high synchrony, but not necessarily with high prosocial emotions (Tschacher et al., 2023). Thus, the relationship between synchrony and pro-sociality definitely depends on context.

Third, it is important to critically consider the pre-processing applied to the times series that serve as the raw data for synchrony computation. Filtering and smoothing of time series change the autocorrelative structure of the processes and thus influence synchrony. Especially if surrogate tests are implemented, using little or no preprocessing appears preferable.

In conclusion, when these (self-)critical points are increasingly heeded in the future, it may be recognized that synchrony research constitutes an implementation of the core of synergetic theory, self-organized pattern formation, with great value for psychology. Synchrony is a fruitful psychological concept also because it interconnects bodily process and mental experience, thus is a prominent example of embodied cognition (Newen et al., 2018). Synchrony research is a hallmark of the integration of complexity theory and Synergetics into the psychology of social interaction.

## Data Availability

The original contributions presented in the study are included in the article/supplementary material, further inquiries can be directed to the corresponding author.
